# Comparison between breathing and aerobic exercise on clinical control in patients with moderate-to-severe asthma: protocol of a randomized trial

**DOI:** 10.1186/1471-2466-14-160

**Published:** 2014-10-17

**Authors:** Karen B Evaristo, Milene G Saccomani, Milton A Martins, Alberto Cukier, Rafael Stelmach, Marcos R Rodrigues, Danilo F Santaella, Celso RF Carvalho

**Affiliations:** Department of Physical Therapy, School of Medicine, University of São Paulo, Av. Dr Arnaldo 455, Rm 1210, São Paulo, SP 01246-903 Brazil; Department of Medicine, São Paulo, SP Brazil; Medicine and Pulmonary Diseases, São Paulo, SP Brazil; Sports Center, University of São Paulo, São Paulo, SP Brazil

**Keywords:** Physiotherapy, Breathing exercise, Aerobic exercise, Symptoms, Asthma control

## Abstract

**Background:**

Asthma is a chronic inflammatory airway disease characterized by reversible obstruction, inflammation and hyperresponsiveness to different stimulus. Aerobic and breathing exercises have been demonstrated to benefit asthmatic patients; however, there is no evidence comparing the effectiveness of these treatments.

**Methods/design:**

This is a prospective, comparative, blinded, and randomized clinical trial with 2 groups that will receive distinct interventions. Forty-eight asthmatic adults with optimized medical treatment will be randomly divided into either aerobic (AG) or breathing exercises (BG). Patients will perform breathing or aerobic exercise twice a week for 3 months, totalizing 24 sessions of 40 minutes each. Before intervention, both groups will complete an educational program consisting of 2 educational classes. Before and after interventions, the following parameters will be quantified: clinical control (main outcome), health related quality of life, levels of anxiety and depression, daily living physical activity and maximal exercise capacity (secondary outcome). Hyperventilation syndrome symptoms, autonomic nervous imbalance, thoracoabdominal kinematics, inflammatory cells in the sputum, fraction of exhaled nitric oxide (FE_NO_) and systemic inflammatory cytokines will also be evaluated as possible mechanisms to explain the benefits of both interventions.

**Discussion:**

Although the benefits of breathing and aerobic exercises have been extensively studied, the comparison between both has never been investigated. Furthermore, the findings of our results will allow us to understand its application and suitability to patients that will have more benefits for every intervention optimizing its effect.

**Trial registration:**

Clinicaltrials.gov; Identifier: NCT02065258.

## Background

Asthma is a chronic inflammatory disorder of the airways that involves many cells and cellular elements. The disease severity can be classified as intermittent or persistent (mild, moderate or severe), and this classification considers the presence of diurnal and nocturnal symptoms, necessity of medication, frequency of exacerbation, physical activity limitations and pulmonary function [1]. The chronic inflammation is associated with airway hyperresponsiveness and airflow obstruction, which lead to recurrent episodes of wheezing, breathlessness, chest tightness and coughing, particularly at night or in the early morning. All of these symptoms deteriorate in the patient’s quality of life and psychological well-being and restrict daily living physical activities (DLPA) [2].

The asthma symptoms experienced during DLPA or the fear of triggering symptoms may keep asthmatic subjects from engaging in physical exercise, and the patients tend to be less physically active and less conditioned than healthy individuals [3]. In addition, asthmatic patients have higher levels of anxiety and depression that have been shown to be associated with an increased number of exacerbations [4] and the diagnosis of severe asthma [5]. These psychosocial disorders can modify the respiratory breathing pattern, which leads to irregular breathing, frequent sighing, and predominant thoracic breathing [6]. These irregular breathing patterns increase the number of respiratory (breathlessness, chest tightness and pain) and non-respiratory symptoms (anxiety, dizziness and fatigue) [7].

Asthma does not have a cure but its clinical manifestations can be controlled with the appropriate treatment. The goal for asthma treatment is to achieve and maintain asthma control, and the treatment is based on medication (controllers and relievers). Clinical control is defined as an effective management of the characteristics of the disease, which include control of the following symptoms: nocturnal awakening, reliever use and activity limitation [8]. However, recent studies have also shown that non-medicinal treatments are important adjuvants in asthma treatment, and the main techniques used worldwide are aerobic [9] and breathing exercises [10].

There is evidence suggesting that aerobic training improves fitness and health related quality of life (HRQoL) [11]. Additionally, aerobic training reduces psychosocial distress [2], exercise-induced bronchoconstriction [12] and corticosteroid consumption [13], exacerbation episodes and asthma symptoms [13]. Moreover, breathing exercises have been shown to improve HRQoL [14] and expiratory peak flow values [15]. Breathing exercises also reduce the levels of anxiety and depression [16], asthma symptoms [17], the use of relief medication [14], exacerbation episodes and airway hyperresponsiveness [18].

The research questions were:

1. Which exercise intervention is more effective to improve clinical control in patients with moderate and severe asthma?
2. Which are the mechanisms involved to improve clinical control in both interventions?

## Method

### Design

**Figure 1:**
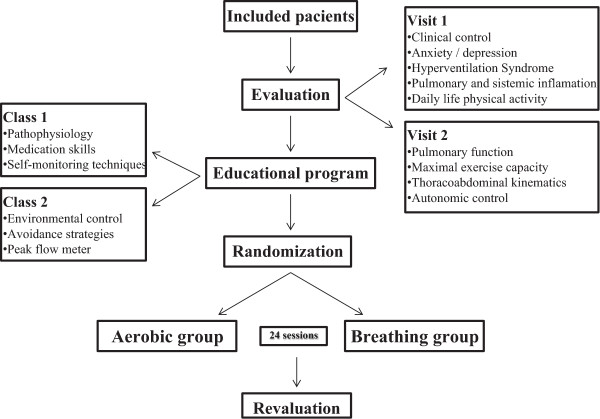
**Study flow chart: after inclusion, patients will be evaluated twice.** In visit one clinical control, pulmonary and systemic inflammation and psychosocial variables will be measured and in visit two pulmonary function, maximal exercise capacity, thoracoabdominal kinematics and autonomic control will be assessed; afterwards subjects will begin the educational program, consisting of two classes: one related to the disease and skills concerning medication and self-monitoring, and other related to control of external factors and peak flow meter usage. Only then, subjects will be randomized into aerobic or breathing group for 24 sessions, after which they will be revaluated.

This trial was designed as a prospective, comparative, blinded, and randomized clinical trial with 2 groups that will receive distinct interventions. See the study design in Figure 1.

### Participants, therapists, centers

#### Study setting

Patients will be recruited from an outpatient clinic that assists moderate and severe asthmatic patients in an University Hospital. The Clinics Hospital Ethics Committee approved the study (0097/10). All participants gave written informed consent before data collection began.

### Allocation and randomization

Eligible patients will be randomized to either breathing or aerobic exercise interventions. The randomization schedule was computer-generated by the chief investigator who will not be involved in the recruitment or treatment of the patients.

### Eligibility criteria

The Hospital Ethics Committee approved the study and patients must provide written informed consent before any study procedures be performed. Asthma diagnosis and treatment will be based on the guidelines of the Global Initiative for Asthma [19].

#### Inclusion criteria

Age between 25 and 65 years-old; both genders; body mass index (BMI) <35 Kg/m^2^; sedentary (currently doing < 60 minutes of structured/planned physical activity per week); medical treatment for at least 6 months; and clinically stable (i.e., no crises or changes in medication for ≥30 days).

#### Exclusion criteria

*S*mokers; participants in another research protocol; patients that are incapable of exercising; unable to understand any questionnaire; any other medical condition that confers greater morbidity than asthma (e.g., active cancer) which will be confirmed by physician review; currently pregnant.

### Intervention

#### Educational program

Before the interventions, both groups will complete an educational program that consists of 2 classes performed once a week and lasting 2 hours each. Presentations and group discussions will include information about asthma pathophysiology, medication skills, self-monitoring techniques, and environmental control and avoidance strategies according to previous studies [2] and asthma guidelines [19].

### Aerobic training program

It will be performed on a treadmill with an initial intensity of 60% and reaching a maximum intensity of 80% of the predicted heart rate during training. The intensity values will be calculated using Karvonen’s formula [20] (1957). Exercise intensity will be increased if the patient does not experience an increase in asthma symptoms during the exercise for 2 consecutive training days. Before and after every session, patients will perform a peak expiratory flow test. If these values are lower than 70% of the patient’s maximum value, then the patient will be advised to use the rescue dose of a bronchodilator prescribed their physician. All of the aerobic training details have been previously described elsewhere [13].

### Breathing exercise

Breathing exercise will be based on Yoga’s breathing techniques [21] and will focus on stimulating nasal and diaphragmatic breathing, increasing the expiratory time, slowing the respiratory flow and regulating the breathing rhythm. Breathing exercises will be divided into 3 phases (lasting one month each) with an intensity progression every 8 sessions (Table 1).

**Table 1:** **Breathing exercise program**

1st phase		
Exercise	Duration	Repetition
*Lying position*		
Raise right arm, left arm and then both, stretching the intercostal muscles	20 s	2
Pull right knee, left knee and then both, stretching paraspinal muscles	20 s	2
Turn hip to the side, keeping your shoulders flat on the mat. One hand holds the knee while lifting up the other arm	20 s	1 each side
Lateral decubitus, one arm supporting the head and other remains draped over the body. Inspiring, rise the arm above the head, eeturning to the starting position, at expiration	1 breath	4 each side
Keep an outstretched arm, while making three deep breaths, return.	20 s	1 each side
Inspire holding knees and pull them toward your chest as you exhale	1 breath	4
With the body length, holding the head with hands and take them towards the chest	20 s	1
*Sitting*		
With your legs extended and stretch the posterior muscles, leading arms outstretched to the feet	20 s	1
Stretch intercostal muscles and obliques, and raising arm laterally flexing the trunk	20 s	2 each side
Introduction to the breathing exercise with hands on abdomen and the diaphragmatic stimulation with active expiration	1 breath	10
Kapalabhati - quick expirations generated by vigorous contraction of the rectus abdominis	1 breath	3 series of 30
With your legs extended and stretch the posterior muscles, leading arms outstretched to the feet	20 s	1
**2nd phase**		
**Exercise**	**Duration**	**Repetition**
*Lying position*		
Raise right arm, left arm and then both, stretching the intercostal muscles	20 s	1
Pull right knee, left knee and then both, stretching paraspinal muscles	20 s	1
Lateral decubitus, one arm supporting the head and other remains draped over the body. Inspiring, rise the arm above the head, returning to the starting position, at expiration	1 breath	4 each side
Keep an outstretched arm, while making three deep breaths, return.	20 s	1 each side
Boat adapted: feet flat, knees bent and hands on his thigh, inhale deeply and take hands toward the knees contracting the abdomen	1 breath	4
Inspire holding knees and pull them toward your chest as you exhale	1 breath	4
Preparation Uddhiyana: arms along the body raise the arms in inspiration, expiration returns	1 breath	4
Preparation Uddhiana: actively inhale and exhale, and apnea raise and lower arms	5 s	4
*Sitting*		
With your legs extended and stretch the posterior muscles, leading arms outstretched to the feet	20 s	1
Preparation Uddhiyana: raise the arms sideways in inspiration and return in expiration	1 breath	4
Preparation Uddhiana: actively inhale and exhale, and apnea raise and lower arms	5 s	4
Uddhiyana Bandha: complete expiration followed by inspiratory effort in the presence of apnea	5 s	1
Uddhiyana Bandha + Kapalabhati	1 breath	3 series of 1 and 45
With your legs extended and stretch the posterior muscles, leading arms outstretched to the feet	20 s	1
**3rd phase**		
**Exercise**	**Duration**	**Repetition**
*Lying position*		
Raise right arm, left arm and then both, stretching the intercostal muscles	20 s	1
Pull right knee, left knee and then both, stretching paraspinal muscles	20 s	1
Lateral decubitus, one arm supporting the head and other remains draped over the body. Inspiring, rise the arm above the head, returning to the starting position, at expiration	1 breath	4 each side
Keep an outstretched arm, while making three deep breaths, return.	20 s	1 each side
Boat adapted: feet flat, knees bent and hands on his thigh, inhale deeply and take hands toward the knees contracting the abdomen	1 breath	4
Inspire holding knees and pull them toward your chest as you exhale	1 breath	4
*Sitting*		
With your legs extended and stretch the posterior muscles, leading arms outstretched to the feet	20 s	1
Uddhiyana Bandha + Kapalabhati	5 s and 1 breath	2 series of 1 and 60
Surya bedhana: inhale through the right nostril, take head towards the chest in apnea returns to the starting position and exhale through the left nostril.	5 s	1 each side
Surya bedhana + Kapalabhati	5 s and 1 breath	2 series of 1 and 70
With your legs extended and stretch the posterior muscles, leading arms outstretched to the feet	20 s	1

All participants will be required to maintain their normal medical regimens during the interventions.

### Outcome measures

#### Primary outcome

The asthma control questionnaire (ACQ) [22] will be used to assess asthma control. We will compare the differences between the 2 interventions in the absolute change in asthma control post-intervention. This questionnaire is validated to measure asthma control in adult patients and have been validated to Brazilian Portuguese [23]. ACQ is a simple instrument and is easy to use that quantifies the following asthma symptoms: nocturnal and morning symptoms, limitation of daily activities, dyspnea and wheezing, and the use of medication (short-acting β_2_-agonist). Patient’s will be scored on a scale ranging from 0 to 6 (0 = without limitation and 6 = maximum limitation). The clinical score of forced expiratory volume (% of predicted, pre-bronchodilator) will be scored on similar scale. The items will be calculated, and the scores of the ACQ will be the average from 7 items between 0 (fully controlled) and 6 (severely uncontrolled). The cutoff will be adopted from previously developed studies, considering controlled (ACQ <0.75 points), not well controlled or poorly controlled asthma (ACQ > 1.5 points). A clinically effective treatment results in a 0.5 point decrease in the score after the intervention [24].

#### Secondary outcomes

##### Psychosocial morbidity

The asthma quality of life questionnaire (AQLQ) [25] and the hospital anxiety and depression (HAD) scale [26] will be used to assess psychosocial morbidity. We will compare the differences between both interventions in the absolute values. AQLQ is a questionnaire divided into the following 4 domains: physical limitation, frequency of symptoms, socioeconomic, and psychosocial. Each domain has a maximum score of 33, 6, 11, and 7 points, respectively that will be converted into percentages, where higher scores represent better HRQoL. The HAD has 14 items divided into 2 subscales (7 for anxiety (HAD-A) and 7 for depression (HAD-D)). For each item, there are 4 alternatives, ranging in score from 0 to 3. The sum of each subscale score ranges from 0 to 21. The cutoff value for anxiety and depression is 8 and 9, respectively.

##### Daily living physical activity (DLPA)

An accelerometer (Power Walker SW 610, Japan) will quantify the absolute change in DLPA. The accelerometer records the total daily number of steps, the number of steps performed at moderate intensity (≥110 steps per minute) and the time spent during DLPA. The assessment will be performed over a period of 7 days, and the DLPA will be quantified before and after the interventions. The accelerometer automatically records the total number of steps during the 7 days, and each patient will record their amount of moderate intensity DLPA in a dairy. The accelerometer will be used to average the number of steps performed during the 5 day period (disregarding the first and last days) [27].

##### Maximal exercise capacity

Shuttle walking test [28] will assess exercise capacity and it will compare the absolute change in maximal exercise capacity post-intervention. Cones set up in a corridor will demarcate 10 meters, and the patients will be asked to walk around the cones at a speed pre-determined by sound signals. The sound signals indicate the moment when the patient must round the cone, and the speed increases at a rate of 0.6 km/h per minute. The total protocol consists of 12 stages, and the test can be discontinued by the individual or the therapist if the patient is unable to maintain the speed required to complete the stage (distance greater than 0.5 m from the cone). The test will be discontinued if the patient present chest pain, intolerable dyspnea, dizziness, cramps, leg pain or pallor. Patient’s heart and respiratory rates, blood pressure, dyspnea and leg fatigue will be measured before and after the test. The scale of perceived exertion [29] will be used to measure dyspnea and leg fatigue.

##### Pulmonary function

It will be evaluated using spirometry (SensorMedics 229; SensorMedics Corp; USA) before and after the inhalation of 200 mg of salbutamol. Technical procedures of spirometry will be performed as recommended by the American Thoracic Society and European Respiratory Society [30]. Predicted normal values will be used as proposed by Pereira et al., [31]. An increase of 12% and 200-mL in FEV_1_ (from baseline) will be characterized as a positive response to the bronchodilator.

### Other notable outcomes – possible mechanisms

#### Hyperventilation syndrome symptoms

Nijmegen questionnaire will be used to assess hyperventilation symptoms. We will compare the differences between the 2 treatments in the absolute change in hyperventilation symptoms post-intervention. The Nijmegen questionnaire is composed of 16 questions that quantify abnormal breathing; (each question range in scale from 0 (never) to 4 (very often)). A total score of ≥23 establishes hyperventilation syndrome with a sensitivity of 91% and a specificity of 95% [32].

#### Autonomic nervous control

Patient’s heart rate variability (HRV) will be assessed at rest pre and post-intervention by a heart rate monitor (Polar S810i, Finland) as previously described [33] and data will be recorded and immediately transmitted to the computer for analysis using the Polar Precision Performance software (release 3.00, Kempele, Finland). HRV parameters will be analyzed according to the components of low frequency (LF), high frequency (HF), LF/HF ratio, standard deviation of the differences between adjacent normal RR intervals (RMSSD) after Fourier transformation and noise filtering through the program Kubios HRV Analysis Software version 2.0 (Kuopio, Finland). Data will be recorded in a 5 minutes interval.

#### Thoracoabdominal kinematics

It will be evaluated using optoelectronic plethysmography (OEP System, BTS, Italy), as previously described [34]. Video is recorded with 8 solid-state charge-coupled cameras operating at 100 frames per second and synchronized with an infrared flashing light-emitting diode. Four cameras will be positioned in front of the subject and four behind. Eighty-nine retro-reflective markers will be placed on the anterior and posterior sides of the trunk according to the protocol previously described [35]. A three-dimensional calibration of the equipment will be performed, based on the manufacturer’s recommendations. The assessment will be performed with the subject seated on a chair without back support and patients will be requested to perform 8 quiet breaths followed by 8 deep breaths guided by blinded therapist. The average of 6 homogeneous respiratory cycles will be used for the data analysis. The chest wall volumes and inspiratory muscular activity will be assessed concurrently. The following variables will be measured: total chest wall and compartmental volumes; time variables and thoracoabdominal asynchrony; activity of the sternocleidomastoid, and external superior and inferior intercostal muscles by surface electromyography (EMG BTS, Italy).

#### Airway inflammation

It will be quantified in collected sputum samples and exhaled fraction of nitric oxide (FE_NO_). Before the sputum collection, subjects will be advised to blow their nose and rinse their mouth with water and swallow it to reduce contamination of the sputum specimen with postnasal drip and saliva. Then, patients will inhale 400 μg of salbutamol followed by a 3% hypertonic saline solution inhaled over 15 min using an ultrasonic nebulizer with an output of 2.4 mL.min^-1^ and a mass median aerodynamic diameter of 4.5 μm as previously described [36]. Sputum samples will be visually separated from the saliva and divided into 2 aliquots. One aliquot will be spread over a glass slide, to be fixed and stained with Diff Quick (Sigma-Aldrich, São Paulo, Brazil). The second aliquot will be treated with 0.1% of dithiothreitol (Sigma-Aldrich, USA) and stirred using a vortex mixer for total and differential cell counts. The total cell counts will be performed using a Neubauer chamber, and the cell suspension will be adjusted to 1.0 × 10^6^.mL^-1^. Differential cells counts will be classified as eosinophils, lymphocytes, neutrophils, macrophages, squamous cells, goblet cells, and ciliated cells on the basis of their morphology by a single-blinded investigator.

*FE*_*NO*_ will be measured according to the ATS/ERS guidelines (2011) [37]. Briefly, patient’s will be asked to blow into a Mylar bag, (keeping the expiratory pressure at the mouth at 12 cm H_2_O to avoid air contamination from the nasal cavity). Exhaled air will be filtrated before being collected into the bag, and the expiratory pressure achieved by the individual will monitored by a manometer. All collected samples will be mixed 10 s before the determination of NO concentration by chemiluminescence (Sievers 280 NOA; Sievers Instruments, USA) and analyzed up to 24 h after sample collection. The equipment will be calibrated before each analysis.

#### Systemic inflammation

It will be quantified in 15 mL of blood sample obtained at rest. Patients will be advised to fast for at least 8 hours not carry out physical activity and not ingest alcoholic and/or caffeinated beverages for 24 hours before the blood collection. Blood samples will be centrifuged at 1800 rpm for 10 minutes at 0°C and will be stored at -70°C. Th1 (IL-6, TNF), Th2 (IL-4, IL-5, and IL-13), and anti-inflammatory (IL-10 e IL-1ra) cytokines as well as osteopontin will be quantified in duplicate by using commercial cytometric bead array kits (BD Biosciences, USA). Serum levels of cortisol will be analyzed by fluoroimmunoassay using the AutoDelfia system (Wallac, Finland).

### Additional measures

To appropriately describe our patients, we will collect basic demographic and medical information including age, smoking status, ethnicity, gender role and anthropometrics (height and weight). It will also be collected self-reported details of current and lifetime history of diagnosis of asthma, other respiratory diseases, cardiovascular disease risk factors, cancers, auto-immune diseases, and all current medications. All reported clinical data will be verified by a hospital medical record review.

### Data analysis

#### Sample size

It was estimated based on a minimal important difference of 0.5 points on the ACQ questionnaire with a standard deviation of 0.72 [38]. A sample size of 40 patients was required for a 5% level of significance with 80% power using a two-tailed *t* test. An estimated drop-out rate of 20% was used to determine the final target sample size of 48 patients.

#### Proposed statistical analyses

Data normality will be evaluated with the Kolmogorov-Smirnov test. The comparison of the clinical control and other variables will be made by a two-way analysis of variance (ANOVA) with repeated measures followed by the Student-Newman Keuls post-hoc test to identify significant differences. The level of statistical significance will be set at 5% (p < 0.05).

#### Analyses population and missing data

All main analyses will use intention-to-treat, considering all patients as randomized regardless of their adherence to the treatment protocol or completion of post-intervention assessments. These analyses will be used to define the efficacy of aerobic and breathing exercises to influence asthma control.

## Discussion

Asthma symptoms have a significant impact on patient’s life and the proper clinical treatment is very important to reduce those symptoms; however, sometimes clinical control is difficult, despite the appropriate amount of prescribed medication. Aerobic and breathing exercises have been considered important as complementary therapies in addition to the pharmacological treatment mainly for patients with controlled or partially controlled asthma, because they improve disease control [1]. In addition, asthmatic patients have 2 main clinical conditions that support the importance of these non-pharmacological interventions: they are more prone to be physically deconditioned compared with their peers and they also have a high prevalence of hyperventilation symptoms [39]. Because of that, asthmatic patients tend to face more negative attitudes toward exercise and present higher levels of anxiety and depression [40, 41].

The proposal of our study, comparing the benefits of aerobic and breathing exercises training on clinical control, seems quite relevant because even if both techniques have similar effects in reducing asthma symptoms, we will further investigate some mechanisms in order to understand how patients develop their benefits at each and every intervention. There is evidence that aerobic exercises improve cardiovascular fitness, resistance to stress and health related quality of life (HRQoL) as well as decrease dyspnea, incidence of exercise-induced bronchospasm, use of corticosteroids, inflammatory parameters, anxiety and depression related to asthma [9]. A recent Cochrane review confirmed these benefits and also demonstrated that physical training can be well tolerated among people with asthma [42]. These studies show us that there was some evidence to suggest that physical training may have positive effects on HRQoL. Therefore, exercise training can also improve daily living physical activity and reduce dyspnea [43]. In our opinion, the improvement in aerobic capacity will reduce airway inflammation levels improving clinical control.

In addition, the practice of breathing exercises have been shown to improve HRQoL, peak expiratory flow, asthma symptoms as well as to reduce the levels of hyperventilation symptoms, anxiety and depression and medication consumption [44]. A recent Cochrane review demonstrated that individual trials reported positive effects of breathing exercises, but authors concluded that no reliable conclusions could be drawn concerning the use of breathing exercises in asthma control [10]. In our opinion, breathing exercise will reduce anxiety and depression levels, which seem to be associated with dysfunctional breathing such as hyperventilation [16] normalizing, thus, the autonomic system [45].

Although the benefits of breathing and aerobic exercises have been extensively investigated, the comparison between both interventions has never been performed. Then, this study will be the first to make this comparison and to investigate several physiological mechanisms which may be triggered either by aerobic training or by breathing exercises. Furthermore, the findings of our results will allow us to understand its application and suitability to patients that will have more benefits for every intervention optimizing its effect.

### Study status

We declare that the study in is ongoing and we are still analyzing data.

## Abbreviations

ACQ: Asthma control questionnaire
AG: Aerobic group
ANOVA: Analysis of variance
AQLQ: Asthma quality of life questionnaire
BG: Breathing group
BMI: Body mass index
DLPA: Daily living physical activities
FE_NO_: Exhaled fraction of nitric oxide
FEV_1_: Forced expired volume in the first second
FVC: Forced vital capacity
HAD: Hospital Anxiety and Depression Scale
HAD-A: Hospital Anxiety and Depression Scale - anxiety
HAD-D: Hospital Anxiety and Depression Scale - depression
HF: High frequency
HRQoL: Health-related quality of life
HRV: Heart rate variability
ISWT: Shuttle Walking Test
LF: Low frequency
OEP: Optoelectronic plethysmography
RMSSD: Standard deviation of the differences between adjacent normal RR intervals.
